# Hepatoprotective efficacy of quinoa seed extract against CCl_4_ ‐ induced acute liver toxicity in rat model

**DOI:** 10.1002/fsn3.4149

**Published:** 2024-04-04

**Authors:** Maria Arshad, Shabana Kousar, Ahmad Din, Muhammad Afzaal, Muhammad Naeem Faisal, Mian Kamran Sharif, Hina Rasheed, Farhan Saeed, Noor Akram, Faiyaz Ahmed, Mahbubur Rahman Khan

**Affiliations:** ^1^ National Institute of Food Science and Technology (NIFSAT), University of Agriculture Faisalabad Pakistan; ^2^ Department of Food Science Government College University Faisalabad Faisalabad Pakistan; ^3^ Food Safety & Biotechnology Lab, Department of Food Science Government College University Faisalabad Faisalabad Pakistan; ^4^ Department of Clinical Nutrition, College of Applied Sciences in Ar Rass Qassim University Buraydah Saudi Arabia; ^5^ Department of Food Processing and Preservation Hajee Mohammad Danesh Science & Technology University Dinajpur Bangladesh

**Keywords:** antioxidants, medicinal plants, oxidative stress, regeneration, Silymarin, super food

## Abstract

The current research explored the possible protective effect of *chenopodium quinoa* extract against CCl_4_ acute liver toxicity in Sprague Dawley rats. Thirty rats were divided into five groups with six rats in each group. CCl_4_ (Carbon tetrachloride) was administered at a dose rate of 2 mL/kg b.w. intra‐peritoneally once a week for 3 weeks. The plant extract was given through oral gavage for a period of 21 days. Group I served as a normal group which was given with basal diet. Group II was referred to as a positive control group and received CCl_4_ 2 mL/kg body weight (i.p.). Group III was the standard treatment group and received 2 mL/kg CCl_4_ (i.p.) and 16 mg/kg body weight (p.o.) silymarin. Group IV was the plant treatment group, which received 2 mL/kg CCl_4_ (i.p.) and 600 mg/kg body weight of quinoa seed extract (p.o.). Group V was the combined treatment group, which received 2 mL/kg CCl_4_ (i.p.) accompanied with a combination of silymarin (p.o.) 16 mg/kg body weight and quinoa seed extract (p.o.) 600 mg/kg body weight. The liver biomarkers were assessed along with histopathological analysis to observe the changes in the liver. The outcome suggested that the treatment, which was given with the combination of silymarin and quinoa seed extract, significantly enhanced the antioxidant levels, reduced the oxidative stress, and restored the liver function as evidenced by biochemical parameters histopathological studies. The hepatoprotective potential may be due to the antioxidant and anti‐inflammatory properties of quinoa seed extract.

## Graphical abstract

The current study was intended to evaluate the possible protective effect of *Chenopodium quinoa* extract against CCl_4_ induced hepatotoxicity.

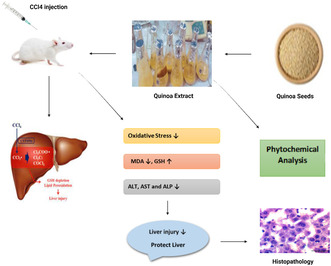

## 1 INTRODUCTION

The liver is a fascinating organ which possesses exceptional characteristics in terms of its anatomy and physiology. It is one of the most important solid glandular organs that comprise about 2% of the body weight of an average adult. In fact, there are no other organs that compete liver with regard to its versatile functions including metabolism, synthesis, immunologic, and hemopoietin. The normal human liver has a great capability to regenerate (Mahadevan, 2020). It is one of the fifth most indispensable organs for human existence that performs vital roles such as bile formation, hormone production, glucose storage, regulating the passage of absorbed substances from the digestive tract, and then distributing to the bloodstream. If there is a complete loss of hepatic function that causes death in seconds, it indicates the significance of the liver (Pannala et al., 2020).

Carbon tetrachloride is a chemical compound with a formula (CCl_4_) (Unsal et al., 2021). It is a widely manufactured chemical as well as a dissolving agent in pharmaceutical medicine. Earlier, it was used in the synthesis of insecticides, shampoos, and refrigerants (Meaden et al., 2020). The CCl_4_ is commonly hepatotoxin employed for inducing liver injury in experimental animals such as rats, mice, and rabbits (Taamalli et al., 2020). It is extremely toxic for the brain, kidney, testis, lungs, and specifically for the liver. The systemic toxicity induced by carbon tetrachloride is due to exposure through ingestion and inhalation. It is a strong toxicant which is dominantly used for inducing toxicity in animal models, particularly in the liver and kidney (Unsal et al., 2021). Its toxicity appears in the form of symptoms like convulsions, weakness, vertigo, and coma. The liver damage may result due to acute exposure after a couple of days whereas kidney damage appears after more than 1 week (Baig & Khan, 2023).

The mechanism of liver injury involves CCl_4_ transferred to free radicals such as trichloromethyl and peroxy‐trichloromethyl radicals through the procedure of biotransformation activated by cytochrome P_450_ that reacts with proteins and DNA causes impairment in the lipid metabolism. Trichloromethyl peroxy radicals are formed by CYP2E1 with the addition of oxygen, causing damage in the cell attached to the protein, nucleic acid, and phospholipid that results in lipid peroxidation occurring in the cell membrane. Prolonged dosage causes huge alterations in the cellular processes that result in inflammation, necrosis, fibrosis, and cirrhosis (Dai et al., 2021).

Globally, the mortality rate of liver diseases is 1.16 million per year and is the 11th major cause of mortality (Asrani et al., 2019). The impairment in liver functions can result in liver injury and eventually lead to organ failure (Wang et al., 2019). The major causes that develop liver diseases are microbes, hepatotoxins, alcohol, overuse of drugs, autoimmune disorder, and inheritable factors (Zhang et al., 2020).

The anti‐hepatitis drugs have become the main reason for liver injury and that is why limiting the use of anti‐hepatitis drugs. On the other hand, there are more than 900 drugs that lead to liver injury, and 20%–40% are observed for fulminant hepatic failure. Consequently, about 75% of idiosyncratic drug reactions are reported in liver transplantation or death (Bourhia et al., 2022). According to the US, there are an estimated 2000 cases of liver failure every year, and more than 50% account due to drugs (Andrade et al., 2019). Drugs can have the ability to either damage directly to hepatocytes or disrupt the flow of bile from the liver. The pathogenesis of drug‐induced liver injury is the disturbance of liver cells, disintegration of transport protein, damage to bile duct, and activation of cytotoxic T cells (Kobayashi et al., 2020).

Herbal medicines have shown better outcomes during the treatment of steatosis. It was evident from the literature that the therapeutic effects of plants are due to the principles encompassing lipid metabolism and inflammation. The plants showed antioxidant activity against liver disease and preventing oxidative stress. Plants contain phytochemicals which indicate numerous pharmacological properties such as anti‐inflammatory, anti‐diabetic, and anti‐cancerous activities (Widodo et al., 2019). They are advantageous for human health because of different biological effects and the ability to control cell signaling pathways. Therefore, in vitro and in vivo studies demonstrated that phytochemicals exhibit promising properties which could alleviate liver diseases (Xu et al., 2020). Liver diseases are the primary concern for the public because orthodox remedies produce many side effects. Herbal medicines have captivated the world's attention toward the development of hepatoprotective agents that are able to protect the liver injury and result in minimal side effects (Ugwu & Suru, 2021).

Quinoa (*Chenopodium quinoa* Willd) is recognized as superfood which is cultivated by Andean region from many decades. Recently, quinoa stood out as one of the excellent grains of the 21st century because of its high nutritional profile. The exceptional therapeutic traits of quinoa contribute to its use as a functional food and nutraceutical. Quinoa has high nutritional profile because of its high content of protein with a balanced amount of essential amino acids. It also comprises a considerable amount of carbohydrates, lipids, and vitamins including vitamins C, E, B2, B6, and B9. In addition, it is also characterized by having an abundant quantity of micro‐minerals. (Wahba et al., 2019).

Quinoa exhibits different pharmacological properties such as antioxidant, anti‐microbial, anti‐diabetic, and immune‐modulatory properties. Previous studies reported that high oxidative stress causes many different ailments in the body including neural disorders, cancer, and Alzheimer disease (Al‐Qabba et al., 2020). The quinoa showed antioxidant activity revealed from many in vivo studies, and its consumption elevated the antioxidant level of enzymes which are superoxide dismutase, glutathione, and catalase along with reducing the oxidative biomarker known as malondialdehyde (Abdel‐Wahhba et al., 2021; Al‐Qabba et al., 2020).

Protein, polysaccharides, and phenolic compounds are present in quinoa which modulate the gut microbiota. The in vitro studies indicated an increase in microbial species such as *Lactobacillus enterococcus* and *Bifidobacterium* that are helpful in maintaining gut homeostasis (Gullon et al., 2016). Short‐chain fatty acids are produced in the body due to the breakdown of non‐digestible polysaccharides as well as change the gut pH and modify the growth of microflora (Fotschki et al., 2020). The anti‐diabetic potential of quinoa is due to the presence of bioactive components including phenolic, protein, tocopherol, and fiber (Liu et al., 2020). The consumption of high protein delays the process of stomach emptying (Cisneros‐Yupanqui et al., 2020). The inhibitory effect of α‐amylase, dipeptidyl‐peptidase‐4, and α‐glucosidase extends the time for insulin secretion to manage blood glucose level (Mudgil et al., 2020).

Inflammation is the natural defense system of the human body to protect from pathogens, but the long‐term gradual inflammation contributes toward autoimmune diseases with weak immunity. Studies have explored that protein, saponins, and phenolic compounds available in quinoa enhanced the immune system (Fan et al., 2019). Furthermore, Capraro et al. (2020) investigated two types of chenopodin which have the potential to down‐regulate the nuclear factor κB activation and IL‐8 expression activated by interleukin 1β. This immune modulatory activity is a trait of a bioactive peptide called lunasin.

The hepatoprotective potential of quinoa is because of its antioxidant and anti‐inflammatory properties. The liver participates in all the metabolic reactions due to which free radicals and oxygen species are generated. Quinoa contains antioxidant components, including phenolic compounds and fat‐soluble vitamins, which attenuate oxidative stress and therefore, may prevent the liver from oxidative damage (Ahmed et al., 2020). The hepatoprotective role of quinoa was evaluated in animal studies reported previously. The quinoa may protect liver functions induced by carbon tetrachloride, high fructose diet, and cyclophosphamide‐exposed rats. This is revealed through a significant decline in liver biomarkers. Consumption of quinoa reduced the level of AST and ALT and is proved to be beneficial for liver homeostasis (Al‐Qabba et al., 2020; Mohamed et al., 2019; Saxena et al., 2017; Wahba et al., 2019). The objective of current research was to determine the hepatoprotective potential of quinoa seed extract in carbon tetrachloride‐induced acute liver injury in Sprague Dawley rats.

## 2 MATERIALS AND METHODS

### 2.1 Procurement of raw material

Quinoa seed was obtained from Virsa Agri farms at the Department of Agronomy, University of Agriculture, Faisalabad Pakistan. The quinoa was sown on the Directorate farms of Agriculture University Faisalabad. The UAFQ7 was the variety of quinoa seed used in research and grown on farms after the approval of Punjab seed council, Federal seed certification, and Registration Department Islamabad, Pakistan. The drug and the reagent used during the study were purchased from Sigma‐Aldrich. However, hepatoprotective drug that was used as a standard drug Silymarin 200 mg bought from nearby Mujahid pharmacy from Jinnah colony Faisalabad, Pakistan. All chemicals used in the current study, including Formalin, Ethanol, Gallic acid, Carbon tetrachloride, Folin‐Ciocalteau reagent, 2,2‐diphenyl‐1‐picrylhydrazyl (DPPH), aluminum chloride, sodium bicarbonate, sodium hydroxide, gallic acid, and chloroform, were procured from Sigma‐Aldrich, St Louis, Mo, USA.

### 2.2 Preparation of hydro‐ethanolic plant extract

**FIGURE 1:**
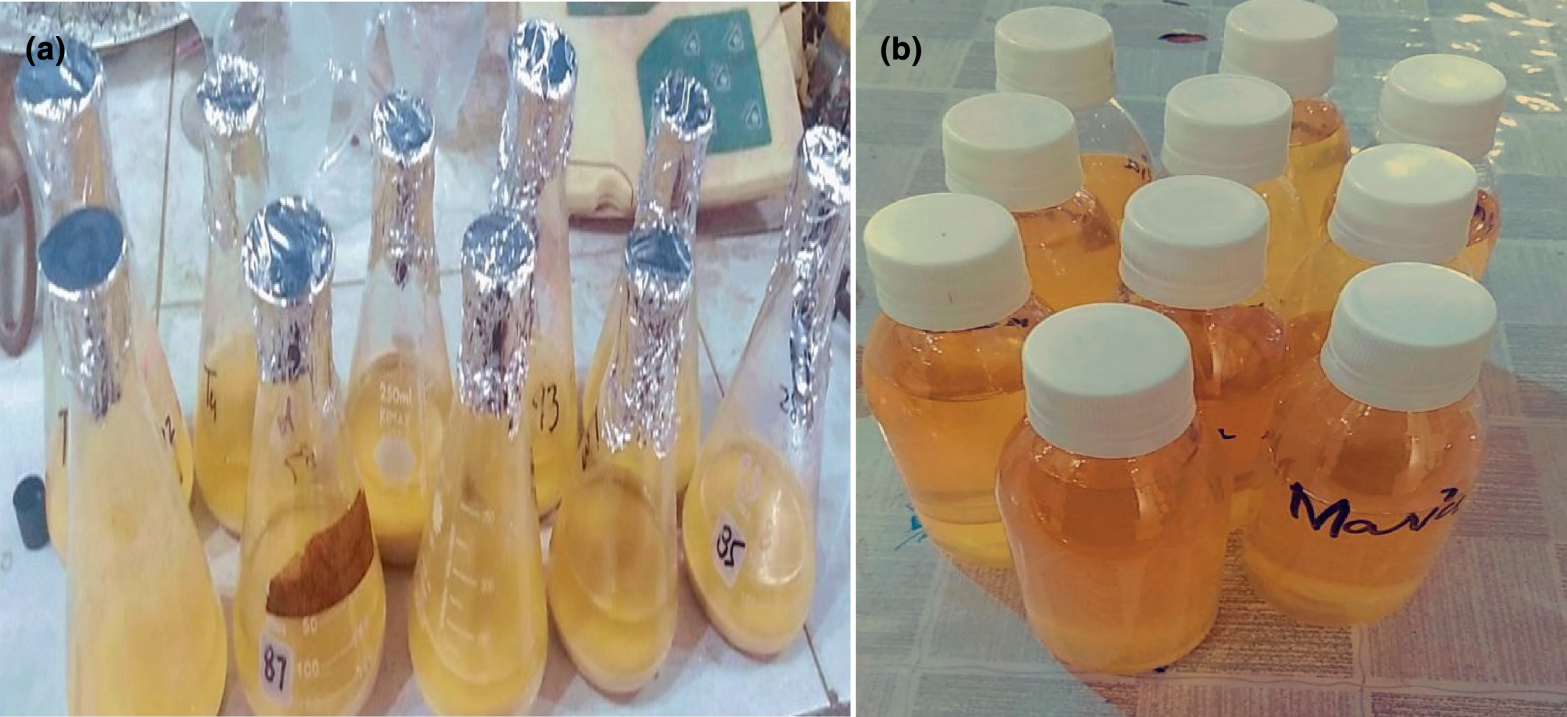
(a) Hydroethanolic extraction of quinoa seed and (b) quinoa seed extract (QSE).

The plant powder was used for extract preparation conducted at the Food Safety Laboratory in the old NIFSAT Department of Agriculture University Faisalabad, Pakistan, by the following method proposed by Miranda et al. (2017). A sample of 10 g quinoa powder was homogenized with 100 mL distilled water and 80% aq. (v/v) ethanol solution. The admixture was placed in orbital shaker for 24 h. Then, after spinning for about 15 min at 5000 g, Whatman N1 filter paper was used to filtrate the supernatant. Then, the filtrate was subjected to rotary evaporator for making it concentrated and lyophilized finally. The extract attained in this process was kept at low temperature (4°C) before its usage. As shown in Figure 1a,b, plant extracts were prepared. The antioxidant analyses (TPC, TFC, and DPPH) were performed in maternal and child nutrition lab home sciences department, University of Agriculture Faisalabad, Pakistan.

### 2.3 Antioxidant analysis

#### 2.3.1 Determination of Total phenolic content (TPC)

TPC was estimated by the following procedure of Folin Ciocalteau reagent with minor changes. About 0.5 mL of aliquot quinoa extract was placed in glass tube, and 0.5 mL of reactive FC and 2 mL of sodium bicarbonate solution was added after 5 min and agitated. The sample was mixed on a vortex mixer and the reaction progressed or continued for 15 min at high temperature. In addition, 10 mL of ultra‐pure water was added to it. The precipitates were removed by the process of centrifugation in 5 min for 4000 *g*. Lastly, the absorbance was estimated by spectrophotometer at 725 nm in comparison to gallic acid calibration curve. Gallic acid standard was used to run against the sample. The result was calculated as gallic acid equivalent in 100 g of quinoa seed on dry weight (Chuah et al., 2008).

#### 2.3.2 Total flavonoid content (TFC)

TFC was measured by aluminum chloride method with slight modification reported by (Carciochi et al., 2015). 2 mL of distilled water was taken and mixed in 0.25 mL of aliquot extract. This was followed by the addition of 0.15 mL of 5% sodium nitrate in the test tube. After 5 min, about 0.15 mL of 10% aluminum chloride solution was added in the admixture. 1 mL of sodium hydroxide was also added to it and diluted the solution with 1.2 mL of distilled water. The absorbance was estimated at 510 nm against blank sample. The result was expressed as catechin equivalent/gram of dry weight.

#### 2.3.3 Free radical scavenging assay

The 2, 2‐diphenyl‐1‐picryl‐hydrazyl (DPPH) radical scavenging assay was used to measure the free radical scavenging activity of the sample with slight modification according to Moncada et al. (2013). The scavenging property of quinoa seed extract was found by adding a 40 μL volume extract in a test tube and 3.960 μL of DPPH stock solution in it. The swirled reaction mixture was mixed for at least 20 s and incubated in dark room for half hour at 151°C. At the end, the absorbance of mixture was recorded at 517 nm by employing spectrophotometer. The stock solution was prepared by using gallic acid (1 mg/mL).

### 2.4 Experimental design

The hepatoprotective impact of quinoa seed extract in carbon tetrachloride liver toxicity was assessed on rat models. The rats were bought from the University of Veterinary and Animal Sciences Lahore Pakistan. Thirty Sprague Dawley (male) rats with a weight of 150–200 g were kept at the Animal House of National Institute of Food Science and Technology, University of Agriculture Faisalabad. All rats were provided with standard conditions including ventilation facility, 12/12‐h period of light dark cycle, and temperature of room about (22 ± 2°C). Prior to the research experiment, ethical approval was obtained from the Office of Research, Innovation and Commercialization. The ethical approval was obtained from the Directorate of Research and Innovation, University of Agriculture Faisalabad (Reference number 1316/ORIC dated 19‐03‐2021). Acclimatization period of one week prior to start the experimental study was provided to the treatment with proper access to normal feed and water. The study was conducted by following the guidelines of animal care of National Biosafety Rules 2005, Punjab Biosafety Rules 2014, and Punjab Animal Act 2019. Rats were divided into five groups and each group contains four rats and placed in designated cages as follows. As showed in Table 1, the intervention groups according to treatment plan have been listed.

**TABLE 1:** Intervention groups of rats based on treatment plan.

Groups	Description
Group I	Normal control (NC) group received basal diet for 21 days.
Group II	Positive control (PC) group received carbon tetrachloride (CCl_4_) intraperitoneal injection (i.p.) 2 mL/kg bodyweight once every week (on 1st, 8th, and 15th day of treatment).
Group III	Treated with CCl_4_ (i.p.) 2 mL/kg body weight as mentioned earlier in addition with the standard drug silymarin (p.o.) 16 mg/kg bodyweight for 21 days.
Group IV	Test group 1 (T1) administered CCl_4_ (i.p.2 mL/kg body weight) as described before in addition to quinoa extract (p.o.) 600 mg/kg bodyweight for 21 days.
Group V	Test group 2 (T2) treated with CCl_4_ (i.p. 2 mL/kg bodyweight) as previously mentioned in addition to the combination of silymarin (p.o.) 16 mg/kg bodyweight and quinoa extract (p.o.) 600 mg/kg bodyweight for 21 days.

**FIGURE 2:**
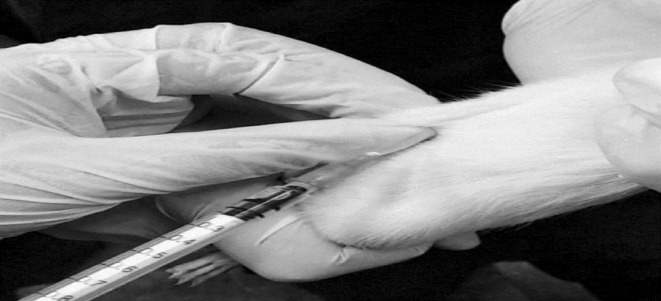
Induction of CCl_4_ (i.p.) to rat for inducing liver injury.

The selected dosage for CCl_4_ injection was (2 mL/kg) by intraperitoneal (i.p.) method to induce liver damage in rats, as shown in Figure 2 (El‐Hashash et al., 2020). Besides it, hepatotoxin (CCl_4_) administered on first, eighth, and fifteenth day of treatment concomitantly with plant extract. Whereas the extract was given as a treatment by oral administration through intragastric tube for 21 days. After 3 weeks of experiment, all rats were decapitated by overnight fasting and using chloroform anesthesia. The blood was collected in heparinized tubes and the serum was separated through the process of centrifugation at 4000 *g* for 20 min. The samples collected for liver function test (ALT, AST, ALP, and total bilirubin) and antioxidant enzyme assay (MDA and GSH) were sent for biochemical assessment to Molecular Care Lab, University of Agriculture Faisalabad.

### 2.5 Biochemical analysis

#### 2.5.1 Determination of ALT and AST

**FIGURE 3:**
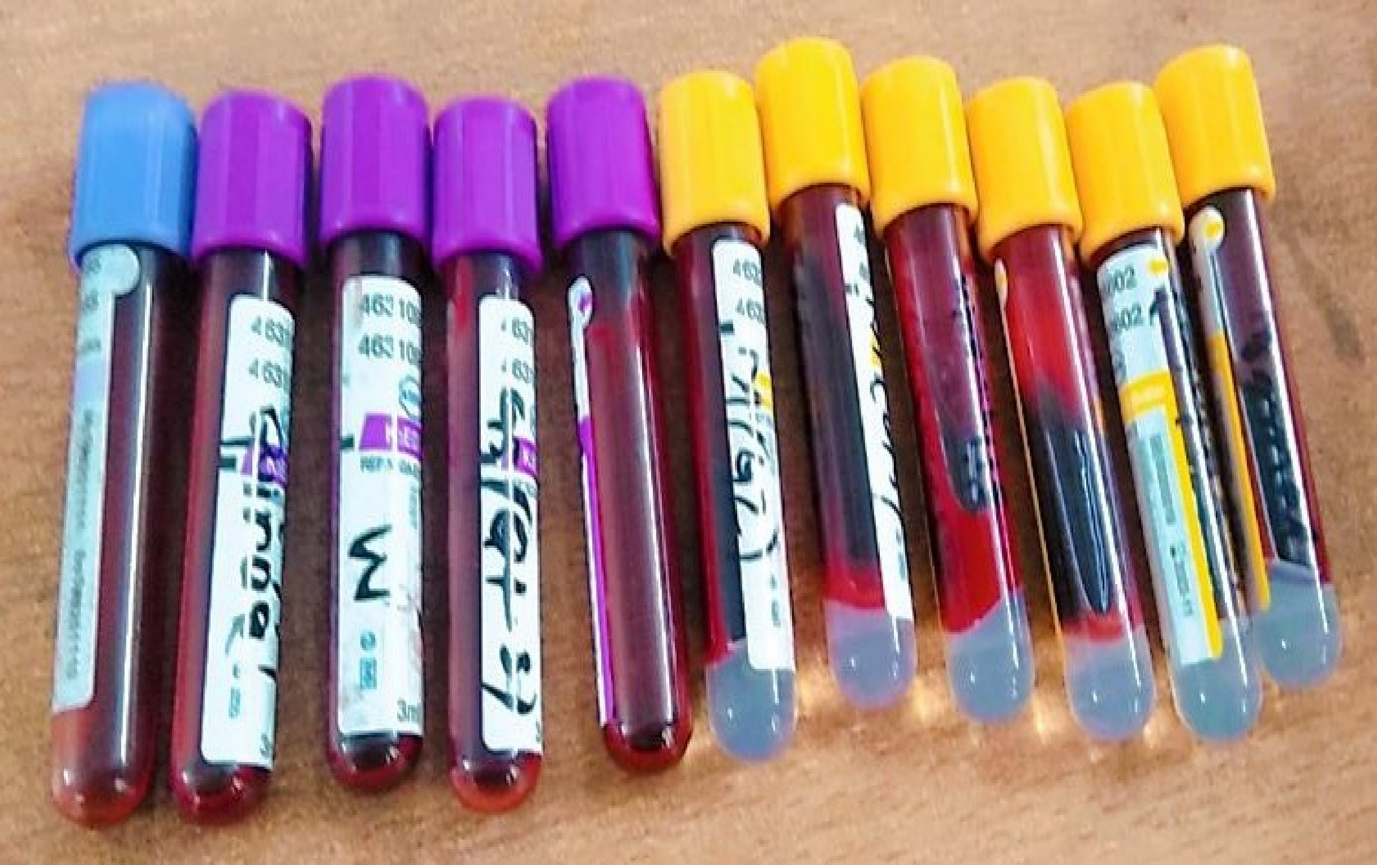
Collection of blood samples for biochemical analysis.

The desired substrate (aspartate and α‐ketoglutarate for AST, alanine and α‐ketoglutarate for ALT) of 1 mL was added into a test tube by using pipette and laid down on water bath for 10 min at uniform temperature of 40°C. Then 0.2 mL of serum was added to mix the material and removed water bath after incubation period of 30 min for GPT and 1 h of GOT. Immediately added the reagent of 1 mL of 2,4‐dinitrophenylhydrazine to cease the reaction. The tube was kept at normal temperature of room for at least 20 min. Then, 10 mL of 0.4 N sodium hydroxide was added to the test tube. The rubber stopper was attached to the test tube and mixed the content by reversion. After half an hour, the water was used as a blank to estimate the optical density of solution at 505 μm by a method of (Reitman & Frankel, 1957). As shown in Figure 3, the blood samples were taken for biochemical analysis.

#### 2.5.2 Determination of ALP

The ALP was measured in IU/L and the method previously performed was followed for determination by (King & Armstrong, 1934). Disodium phenyl phosphate was used as a substrate in the procedure of ALP. The buffer and the substrate were pre‐incubated for 10 min and then added serum of 0.2 mL followed by incubation for 15 min at a temperature of 25°C. The substrate released phenols reacted with 1 mL of folin phenol reagent. The supernatant was collected after the process of centrifugation of suspension. 2 mL of sodium bicarbonate was put in the supernatant and after 10 min the color appeared at 680 nm.

#### 2.5.3 Serum bilirubin

The calorimetric diagnostic method was used to estimate the total bilirubin by the procedure of Malloy and Evelyn (1937). The purple color is important to determine the overall concentration of bilirubin observed in serum for unconjugated bilirubin. After 5 min, the concentration of purple was recorded again at 540 nm by adding methanol for conjugated bilirubin. The net bilirubin is the sum of conjugated and unconjugated bilirubin, and it is represented as mg/dL.

#### 2.5.4 Malondialdehyde (MDA)

The thiobarbituric acid (TBA) was used for the measurement of MDA to determine the concentration of lipid peroxidation in liver by Uchiyama and Mihara (1978). The 0.5 mL of tricholoacetic acid (TCA) and liver homogenate were added together. The suspension was passed through the process of centrifugation for 10 min at 3500 *g*. Took 1 mL of supernatant and 1 mL TBA were added and then the contents were heated on water bath for 10 min. Afterwards the solution was placed on cold bath. The absorbance of mixture was estimated at 535 nm. The result of MDA is expressed as μmol/g.

#### 2.5.5 Glutathione (GSH)

The glutathione can be estimated by following the method of Beutler et al. (1963). In this process, an Eppendorf tube was taken and added 200 μL of sample. The sample was mixed with 300 μL of pure water and 300 μL of precipitating solution in the tube. The precipitating solutions contain sodium chloride, EDTA, and glacial meta phosphoric acid in concentrations of 30, 0.2 and 1.67 g. The solution was subjected to incubation for the first 5 min and then passed through centrifugation for the next 10 min. Further 200 μL of supernatant was added in cuvette and along with it 800 μL phosphate solution and 100 μL DNTB. The absorbance was measured at 412 nm opposed to blank solution.

### 2.6 Histopathology of liver tissue

The following steps were used to perform histopathological analysis. Briefly, the rats were dissected to remove the liver after the completion of experiment, the liver was removed from each animal, washed with normal saline, and preserved in 10% formalin for fixation. The samples of liver were cut into small section of about 5 μm for post fixation. The tissue samples were kept at different concentrations of ethanol, xylene, and paraffin for dehydration and cleaning. The specimen was embedded in paraffin wax to solidify, and the blocks were preserved in refrigeration to avoid contamination. The samples were cleaned with xylene to perform sectioning. Mayer's egg albumin was used to fix the sample on the glass for mounting. The tissue was stained with the help of hematoxylin and eosin for conducting staining (Slaoui et al., 2017). The liver samples were sent to Pathology Department, Faisalabad Medical University, Faisalabad, Pakistan, for histopathological studies (Lab slides no: 3139). The slides were observed under light microscope in the Pharmacology and Physiology departments, University of Agriculture, Faisalabad.

### 2.7 Statistical analysis

The results of all parameters were stated as mean with standard deviation (mean ± SD). By using one‐way analysis of variance (ANOVA), the data were statistically analyzed. Tuckey's test was applied among different treatment groups to make a comparison and to identify the statistical difference (Montgomery, 2017).

## 3 RESULTS

### 3.1 TPC, TFC, and DPPH activity

Polyphenols are the diverse secondary plant metabolites which are present in foods of plant origin. The phenolics and flavonoids are bioactive compounds which possess antioxidant activity. The TPC, TFC, and DPPH of quinoa extract are represented in Table 2. Our study explored that the higher concentration of TFC and the lower concentration of TPC was observed in quinoa seed extract. The difference in phenolics and flavonoids content might be due to extraction techniques and solvents used. Also, several other factors which affect polyphenolic compounds are plant variety, maturity, and environmental conditions. The present data found out that the radical scavenging activity of quinoa seed extract may be due to phenolics and flavonoids. The antioxidant capacity of any compound depends upon the plant genotype, developing circumstances, season, and environmental factors.

**TABLE 2:** TPC, TFC, and DPPH of quinoa seed extract.

Parameter	Concentrations
TPC (mg GAE/g of dry extract)	27.8 ± 0.83
TFC (mg CAE/g of dry extract)	37.4 ± 1.12
DPPH radical scavenging effects %	14.25 ± 0.43

*Note*: All results were performed in triplicate manner with standard deviations. Results have been presented in Means ± SD.

### 3.2 Liver enzymes

**FIGURE 4:**
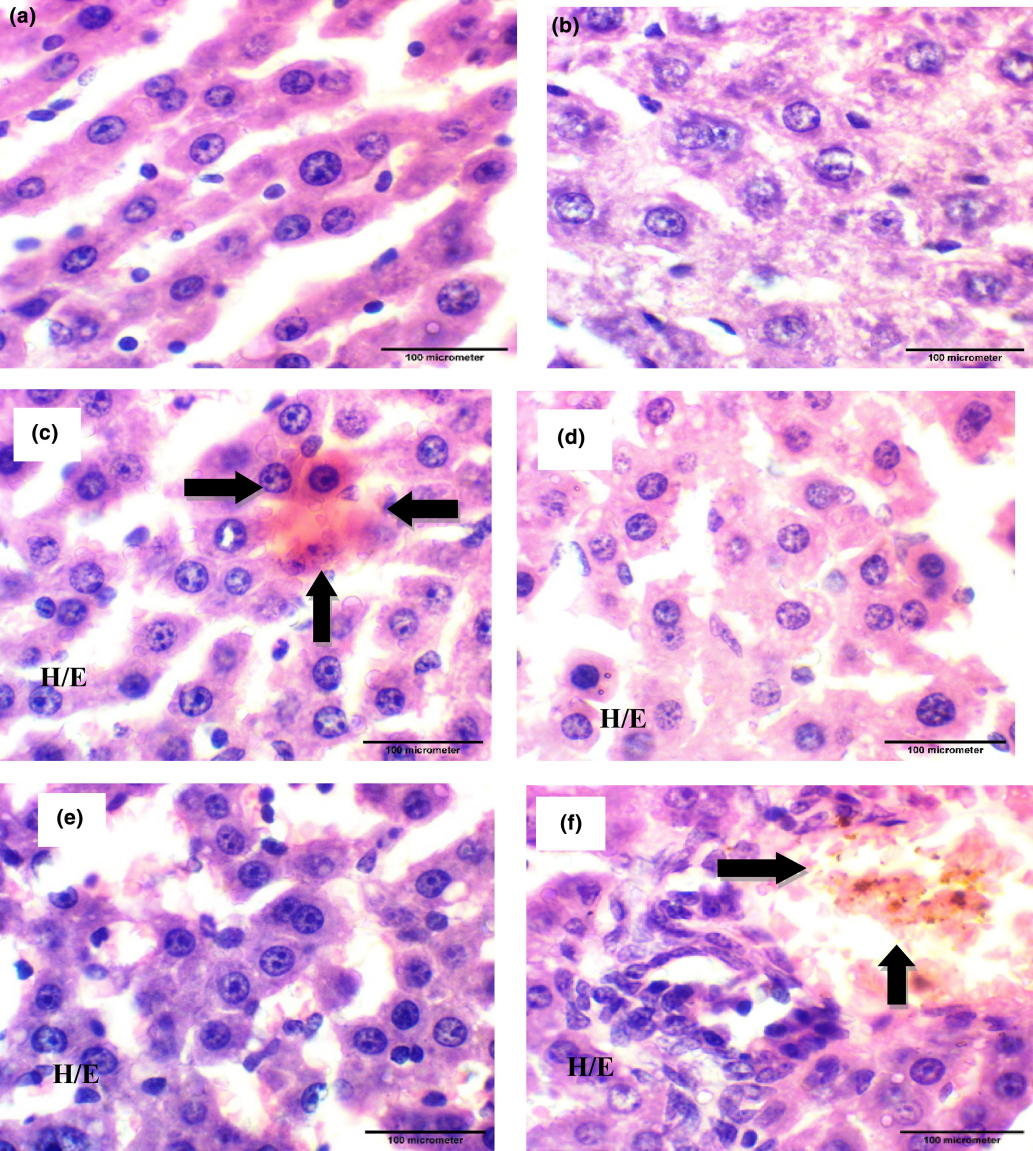
Photomicrographs showing histopathology of liver tissue (H&E staining; 1000×); a photomicrograph of rat liver tissue from groups: (a) Normal Control group; (b, f) Positive Control group received 2 mg/kg CCl_4_ (i.p.); (c) Standard drug group administered with SLM 16 mg/kg p.o.; (d) T1 administered with 600 mg/kg QSE p.o.; (e) T2 received p.o. 16 mg/kg SLM + QSE p.o. 600 mg/kg; (f) CCl_4_ group 2 mL/kg (i.p.). Destruction of hepatocytes and displacement of nucleus in CCl_4_ group while both treatment groups showed tissue recovery with regeneration of hepatocytes.

The outcome of administration of quinoa seed extract was observed in rats after 21 days of treatment. The liver markers are an indication of hepatic damage, and a significant increase was evaluated in liver enzymes (ALT, AST, ALP, bilirubin) in CCl_4_ group in contrast to normal control group as shown in Table 3 and Figure 4. Moreover, both SLM and QSE led to a considerable decrease in the ALT, AST, ALP, and serum bilirubin levels when used alone which establishes the hepatoprotective potential. However, among the treatment group (SLM + QSE), when intervention administered in combination the levels were markedly ameliorated near to normal range and statistically significant (*p* > .01) showing that both have liver protective effects.

**TABLE 3:** MDA, GSH, and serum liver markers.

Groups	MDA (nm/mg)	GSH (nmol/g)	ALT (IU/L)	AST (IU/L)	ALP (IU/L)	Bilirubin (mg/dL)
Normal group	0.55 ± 0.02^d^	9.40 ± 0.23^a^	27.4 ± 0.66^e^	94.79 ± 1.7^e^	60.50 ± 1.45^e^	0.79 ± 0.01^c^
CCl_4_ group	1.48 ± 0.04^a^	5.30 ± 0.16^e^	150.7 ± 2.26^a^	213.73 ± 1.60^a^	180.57 ± 1.91^a^	1.57 ± 0.03^a^
SLM group	0.82 ± 0.02^b^	6.40 ± 0.19^d^	68.00 ± 2.38^b^	167.50 ± 1.80^b^	114.0 ± 1.71^b^	0.92 ± 0.03^b^
Test group 1 (QSE)	0.70 ± 0.02^c^	7.10 ± 0.25^c^	55.00 ± 1.92^c^	149.07 ± 1.90^c^	89.00 ± 1.87^c^	0.89 ± 0.03^b^
Test group 2 (SLM + QSE)	0.65 ± 0.02^c^	8.20 ± 0.29^b^	39.20 ± 1.37^d^	108.80 ± 1.31^d^	67.00 ± 1.0^d^	0.68 ± 0.02^d^

*Note*: MDA (*p* > .01) among different treatment groups and GSH (*p* < .01), among treatments groups compared to normal control group. The result showed that quinoa seed extract and silymarin has significant effect on the level of ALT, AST, ALP, and serum bilirubin (*p* > .01) are shown in superscript letters. Normal control (NC); Positive control (PC) (CCl4 2 mL/kg; IP); SLM: CCl4 (2 mL/kg; IP) + Silymarin (16 mg/kg, p.o.); T1: CCl4 (2 mL/kg; IP) + QSE (600 mg/kg; p.o.); T2: CCl4 (2 mL/kg; IP) + Silymarin (16 mg/kg, p.o.) + QEE (600 mg/kg; p.o.).

### 3.3 MDA

Our result revealed that administration of CCl_4_ alone caused a significant elevation in MDA in positive control group compared to normal control group. Whereas the combine therapy of (SLM 16 mg/kg + QSE 600 mg/kg) reduced the level of MDA induced by CCl_4_ treatment by suppressing the oxidative stress and inflammation. The present study analyzed the data were statistically highly significant (*p* > .01) among different treatment groups (Table 3).

### 3.4 GSH

The research trial also explored that the GSH content was decreased (5.30 nmol/g) in rats following the hepatic injury due to CCl_4_ exposure in comparison to normal control group. The SLM and QSE alone also improved the GSH content to some extent in comparison to CCl_4_ group. Additionally, the hepatic GSH content was significantly raised as (*p* > .01) in group treating with SLM accompanied with QSE through scavenging of free radicals, improving antioxidant enzyme and thereby reducing the liver damage (Table 3, Figure 4).

### 3.5 Findings of histopathological analysis

Histopathology is the study of diagnosis of tissues to examine the alterations under light microscope. The images described the rate of progression and disease control. Figure 4a shows the image of a healthy liver. There was no histological alteration; the section of liver represents the normal morphology with no swelling, necrosis, and cellular infiltration in the control group that received no treatment and induction of liver injury. Figure 4b,f indicates inflammation, necrosis, destruction of hepatocytes, and displacement of nucleus in positive control group that received 2 mL/kg bodyweight of CCl_4_ (i.p.). The SLM group (p.o.) 16 mg/kg body weight which indicated the mild congestion and minimum inflammatory cells in liver cells is shown in Figure 4c. The test group 1 in Figure 4d administered with QSE p.o. 600 mg/kg group decreased the inflammation to moderate level and fatty degeneration. The T2 group treated with the combination of (p.o.) SLM 16 mg/kg body weight and therapeutic agent QSE (p.o.) 600 mg/kg body ameliorated the inflammation of liver cells, lowered the necrosis region, and reduced the inflammatory cells which showed improvement of hepatic function indicated in Figure 4e.

## 4 DISCUSSION

The human body contains the largest organ, which is the liver. It plays several vital functions in the body such as metabolism, removal of toxic substances, and maintenance of homeostasis. There are some major causes of liver damage which are alcohol, environment chemicals, and drugs. Liver damage occurs due to hepatotoxic chemicals which increase oxidative stress, lipid peroxidation, and liver enzymes (Ouassou et al., 2021). Liver diseases have become a major health concern for the public and are responsible for one million deaths each year (Xiao et al., 2019).

**FIGURE 5:**
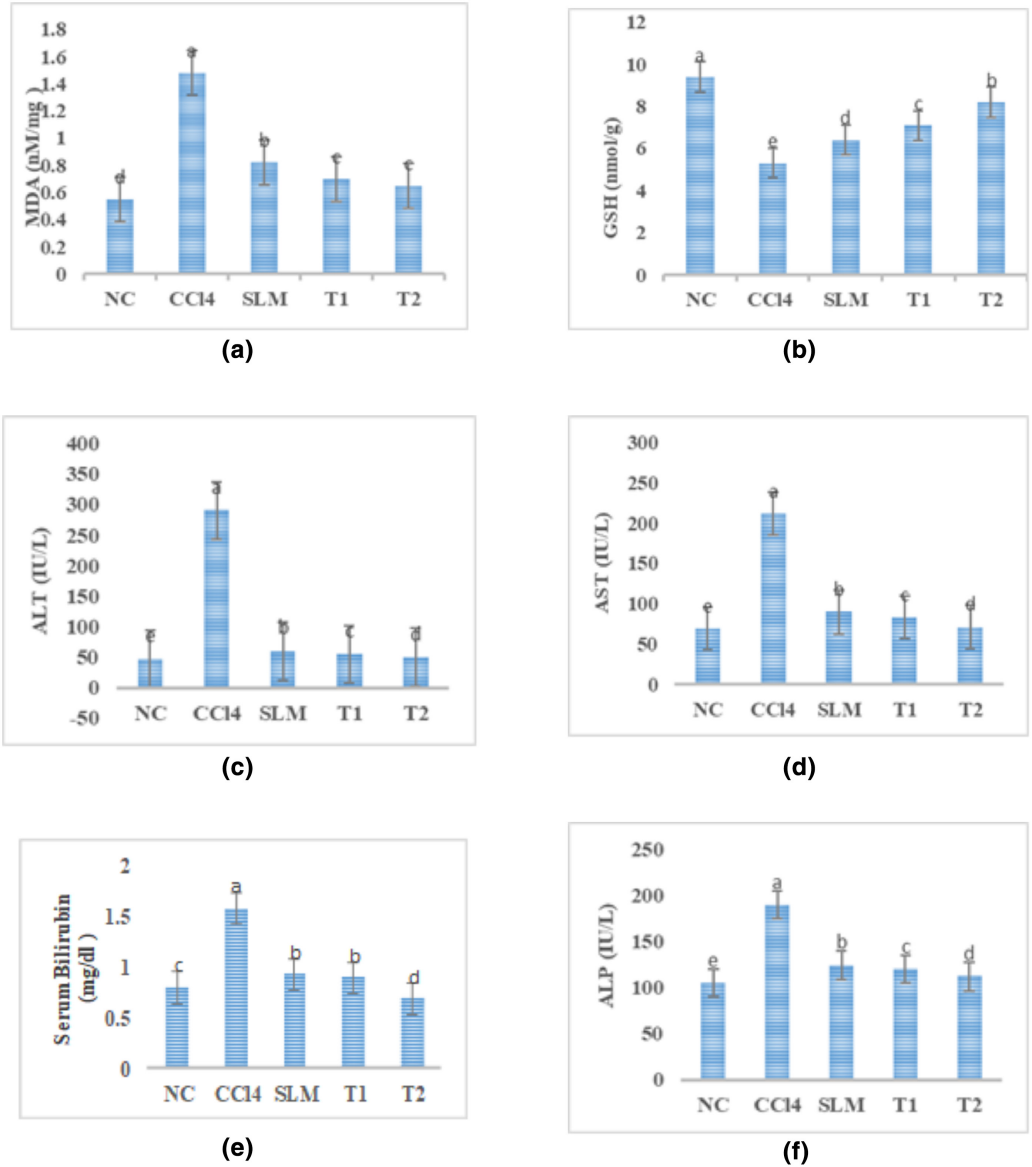
(a) MDA (*p* > .01) among different treatment groups. (b) GSH (*p* < .01) among treatments groups compared to normal control group. The result showed that quinoa seed extract and silymarin have significant effects on the level of ALT, AST, ALP, and serum bilirubin (*p* > .01) shown in c, d, e, and f. Normal control (NC); Positive control (PC) (CCl4 2 mL/kg; i.p.); SLM group: CCl4 (2 mL/kg; i.p.) + Silymarin (16 mg/kg, p.o.); T1 group: CCl4 (2 mL/kg; i.p.) + QSE (600 mg/kg; p.o.); T2 group: CCl4 (2 mL/kg; i.p.) + Silymarin (16 mg/kg, p.o.) + QEE (600 mg/kg; p.o.)

CCl_4_ is commonly used to study hepatoprotective activity in animal models, especially mice, rats, and rabbits. The oxidative stress and lipid peroxidation are the common biomarkers for causing liver damage. The pathology involves huge alterations in the cellular processes that result in inflammation, necrosis, fibrosis, and cirrhosis (Dai et al., 2021). There are currently few treatment options for hepatocellular carcinoma. Among these, chemotherapy, radiotherapy, and transplantation have poor long‐term results and restricted effectiveness. For the development of new innovative drugs, plants have been used as a natural resource owing to their remarkable benefits and less toxic effects (Fan et al., 2022; Yang et al., 2019; Figure 5).

The advanced medicines employed for the treatment of liver ailments have short‐ to long‐term adverse effects such as vomiting, tiredness, xerostomia, constipation, inflammation, and death. The analysis of medicinal plants investigates safer curative or healing effects from natural origin is the area of great concern (Khan Hira et al., 2019). Medicinal plants have been used globally as conventional medicines for the treatment of different ailments from ancient times. There are some studies indicating that phytochemicals can be developed as a substitute for artificial medicines to cure health issues. Some plants have been shown to have promising therapeutic effects against liver problems (Almatroodi et al., 2020).

Quinoa was well recognized as the mother of all the grains. It can be utilized as food or medication for the past 5000 years in the Andean region. The year of 2013 was stated as “The International Year of the Quinoa” by Food and Agriculture Organization (FAO). FAO announced quinoa as a food with complete nutritional characteristics, high diversity, and to accomplish the food security internationally (Alandia et al., 2021). It is worthwhile for controlling many disorders including cardiovascular disease, celiac disease, and non‐alcoholic fatty liver disease (Mohamed et al., 2019; Pourshahidi et al., 2020; Zevallos et al., 2014).

The research aimed to evaluate the hepatoprotective effect of QSE against liver damage. The study was performed by using carbon tetrachloride‐induced liver toxicity in rat's model. Our findings showed that CCl_4_‐treated group elevated the serum liver enzymes level in positive control group compared to other treatment groups. The outcome of present research is also supported by the earlier findings of Shahzad et al. (2021) and Saxena et al. (2017). The structural integrity of liver was affected due to CCl_4_ exposure in rats. Hepatic enzymes are cytoplasmic in nature and leak into the circulation in case of liver damage (Ouassou et al., 2021).

Silymarin was extracted from milk thistle plant, and it consists of three flavolignans which are its active components, namely, silybin, silychristin, and silydianin. It is employed for the purposes of liver diseases and is commonly known as hepatoprotective agent. It has the potential to scavenge the free radicals and provide stabilize effect to cytoplasmic membranes. In experimental trials, a lot of hepatotoxic substances are used for disease induction such as carbon tetrachloride, thioacetamide, and d galactosamine. SLM has a protective activity including ant‐inflammatory, anti‐fibrotic, and immunomodulatory effect (Gillessen & Schmidt, 2020). The study also observed that rat treated with SLM, QSE, and combination therapy of both showed alleviation in the serum level of liver enzymes, showing hepatic cell regeneration or preserve the structural integrity of hepatic membrane (Shahzad et al., 2021; Subramaniam et al., 2015).

Malondialdehyde is a highly reactive species and produced because of peroxidation of fatty acid through the metabolism of arachidonic acid. It is a common biochemical marker for oxidative stress (Arauz et al., 2016). The present study demonstrated that hepatoxicity induced by CCl_4_ raised the inflammation and the oxidative stress as indicated by elevated level of MDA in positive control group in comparison to other treatment groups (Elbakry et al., 2019). Glutathione is the major biomarker of oxidative stress to maintain the redox balance in intracellular. GSH converted to its oxidized form which is glutathione peroxidase (GSSH) by participating in redox reactions through oxidation of thiol group. The level of GSH was reduced because of cell damage due to production of free radicals (Arauz et al., 2016).

In vivo research determined that the SLM, T1, T2 groups improved the GSH content to some extent compared to positive control as shown by suppressed the level of lipid peroxidation and raised the antioxidant enzymes (Pasko et al., 2010; Saxena et al., 2017; Shahzad et al., 2021). The study also found that SLM accompanied with QSE reduced the lipid peroxidation indicated by low MDA and enhanced the antioxidant defense system confirmed by elevated level of antioxidant enzyme (GSH) in CCl4 damaged rats (Al‐Qabba et al., 2020). The flavonoid and phenolic content of hydroethanolic extract of quinoa seed has been presented in Table 1. The contents of TPC, TFC, and DPPH were found to be 27.8 GAE/g of dry weight, 37.4 mg CAE/g dry weight, and 14.25% respectively. The findings of the current study were different from previous studies due to differences in many factors including variety of origin, extraction techniques and harvesting conditions etc. reported similar findings to some extent by Aguilar et al. (2019) and Rahimi and Bagheri (2020).

The current findings confirmed that quinoa has remarkable hepatoprotective property because of the bioactive compounds including phenolics and flavonoids which reduced the oxidative stress reported by Ahmed et al. (2020). The phenolics and flavonoids in quinoa showed diverse pharmacological and biological characteristics including antioxidant, anti‐inflammatory, and free radical scavenging activities (Rahimi & Bagheri, 2020). These bioactive compounds have antioxidant and anti‐inflammatory properties that decreased the lipid peroxidation, scavenged the free radicals, and lessened the inflammation because QSE has been used as a medicinal therapy because of its bioactive potential (Abdel‐Wahhab et al., 2021; Ahmed et al., 2020).

There are also some studies on quinoa against liver fibrosis and damage. The red quinoa bran was assessed against carbon tetrachloride induced (CCl_4_) liver fibrosis. Forty male mice were taken and divided into six groups containing five in each group. First BABL/c mice were administered with CCl_4_ injection in the region of peritoneum to cause liver fibrosis and treatment was performed by providing the diet with red quinoa seed powder, ethanol extraction of bran, water extract, and rutin. It was found that the overall effect of red quinoa powder produced more significant change than rutin in fibrosis and preserved the liver by inhibiting the route of inflammatory markers like tumor necrosis alpha and transforming growth factor beta 1 activities (Lin et al., 2019).

Another study conducted on hepatoprotective efficacy of quinoa seed extract liver damage. CCl4 (1 mL/kg) was administered in rats through intraperitoneal for inducing liver injury. Wistar rats divided into five groups referred as negative control with normal diet, positive control with CCl_4_ 1 mL/kg, standard drug with Silymarin 30 mg/kg, and ethanolic quinoa extract at 400 or 600 mg/kg administered as treatment. The outcome of this research showed that quinoa ethanolic extract prevented liver damage by lowering the level of serum liver markers and supported by gene expression analysis (Shahzad et al., 2021).

## 5 CONCLUSION

The present study shed light on the regenerative role of quinoa seed extract induced by CCl_4_ toxicity in rats. The CCl_4_ toxicity produced radical oxygen species prompting oxidative stress. Our finding suggested that the QSE ameliorated the level of serum liver enzymes near to normal value, enhanced the antioxidant level, and restored the liver functioning in exposed rats. Hence, the quinoa seed extract has capacity to regenerate liver observed in rat model. The phenolic and flavonoid compounds present in QSE offer hepato‐protection which have been attributed to their antioxidant and anti‐inflammatory potential. More studies are needed to know the underlying mechanism of hepatoprotective effect of QSE because it is a mixture of bioactive compounds present and there may be more than one mechanism involved in it.

## AUTHORS CONTRIBUTIONS

**Maria Arshad:** Conceptualization, Investigation, original draft, validation, writing, editing, visualization, and final reviewing. **Shabana Kousar:** Investigation (Supporting), reviewing. **Ahmad Din:** Supervision, reviewing. **Muhammad Naeem Faisal:** Supervision (equal), final reviewing. **Muhammad Afzal:** Final reviewing. **Mian Kamran Sharif:** Supervision. **Noor Akram**: Editing, Writing, Reviewing. **Faiyaz Ahmad, Hina Rasheed** and **Farhan Saeed:** Visualization. **Mahbubur Rehman Khan:** Visualization, Editing, Final Reviewing.

## CONFLICT OF INTEREST STATEMENT

The authors declare no conflict of interests.

## Data Availability

Adequate data has been given in the form of tables, figures, however if more data is required, data will be provided on request basis.
